# Neurodegeneration, Oxidative Stress, NGF/TrkA/P75^NTR^, and PGE2 Dysregulation Induced by PFOS Single and Repeated Treatment: Partial Protection by T3 and Other Therapeutic Approaches

**DOI:** 10.3390/pharmaceutics18030292

**Published:** 2026-02-27

**Authors:** Paula Moyano, Andrea Flores, Javier Sanjuan, José Carlos Plaza, Lucía Guerra-Menéndez, María Victoria Naval, Luisa Abascal, Olga Mateo-Sierra, Javier del Pino

**Affiliations:** 1 Department of Pharmacology and Toxicology, Veterinary School, Complutense University of Madrid, 28040 Madrid, Spain; 2 Department of Legal Medicine, Psychiatry and Pathology, Medicine School, Complutense University of Madrid, 28041 Madrid, Spain; 3 Department of Basic Medical Sciences, Medicine School, Universidad San Pablo-CEU, CEU Universities, Urbanización Montepríncipe, 28660 Boadilla del Monte, Spain; 4 Department of Pharmacology, Pharmacognosy and Botany, Complutense University of Madrid, 28040 Madrid, Spain; 5 Department of Surgery, Medicine School, Complutense University of Madrid, 28040 Madrid, Spain

**Keywords:** perfluorooctane sulfonic acid, basal forebrain cholinergic neurons, thyroid hormones, oxidative stress, NGF/TrkA signaling, PGE2 signaling, neurodegeneration

## Abstract

**Background/Objectives**: Perfluorooctane sulfonic acid (PFOS), a persistent industrial chemical, has been associated with impairments in cognition. While several studies have attempted to identify the underlying mechanisms, the precise pathways mediating these cognitive deficits remain incompletely understood. PFOS induces cell death in basal forebrain cholinergic neurons (BFCNs), a population critically involved in maintaining cognitive function, partially through the disruption of thyroid hormone signaling. These neurotoxic effects could be mediated through multiple interconnected pathways, including the generation of oxidative stress, dysregulation of prostaglandin E2 (PGE2) signaling, and disruption of nerve growth factor (NGF) homeostasis, all of which have been independently linked to BFCN degeneration and cognitive dysfunction and reported to be induced after PFOS exposure. **Methods**: To systematically evaluate PFOS-induced neurodegeneration in BFCNs, we employed the SN56 cholinergic cell line derived from the basal forebrain. Cells were exposed to PFOS across a concentration range (0.1–40 μM) in combination with various pharmacological agents: triiodothyronine (T3; 15 nM), recombinant NGF (20 μM), MF-63 (1 μM), and N-acetylcysteine (1 mM). **Results**: Our experimental results show that PFOS exposure (both single 1-day and repeated 14-day treatments) triggers oxidative stress through reactive oxygen species accumulation coupled with diminished NRF2 pathway activity. Furthermore, PFOS disrupts both PGE2 signaling and the NGF/TrkA/P75^NTR^ neurotrophic pathways, ultimately leading to BFCN cell death. These neurotoxic effects appear to be partially mitigated through T3 treatment, among other mechanisms. **Conclusions**: These findings provide valuable mechanistic insights into PFOS-induced BFCN neurodegeneration and the consequent cognitive decline while simultaneously suggesting potential therapeutic strategies to counteract these detrimental effects.

## 1. Introduction

Perfluorooctane sulfonic acid (PFOS), one of the predominantly employed per- and polyfluoroalkyl substances (PFAS) [1,2,3], is a highly persistent organic pollutant [4]. While regulatory bans in Europe and North America have restricted the use of PFOS due to demonstrated risks, it continues to be utilized in certain regions [4]. Its extreme stability has led to widespread environmental contamination [4], with detectable levels found in wildlife and human biological samples including blood and serum [5]. Compared to other persistent organic pollutants (POPs), such as perfluorooctanoic acid (PFOA), phthalates, or many polychlorinated biphenyls (PCBs), PFOS stands out due to its high environmental persistence (∼5.4 years half-life in humans) [6,7], its ability to accumulate in neural tissues [8,9], its greater neurotoxic potency in vitro compared to other PFAS [10], and the epidemiological association between serum PFOS levels and cognitive impairment in humans [11], justifying its study as a model of environmental pollutant neurotoxicity. At the cellular level, PFOS acts as an endocrine disruptor [5], affects nuclear receptors such as the peroxisome proliferator-activated receptor α (PPAR-α) [12], induces oxidative stress [2,13,14], alters inflammatory signaling, including prostaglandins [15,16,17,18], and promotes apoptosis through multiple pathways, including the activation of p53 [3] and release of mitochondrial DNA, all mechanisms that are related to the induction of neurodegeneration [19,20,21]. Neuropathological research has identified PFOS accumulation in the brain tissue of Alzheimer’s disease patients [22]. Experimental studies in animal models further support these findings, demonstrating that PFOS exposure disrupts learning and memory processes [23]. However, the precise molecular and cellular mechanisms underlying these neurotoxic effects remain unclear.

Previous studies have linked PFOS-induced cognitive dysfunction to neurodegeneration in the frontal cortex and hippocampus in animal models [23,24]. The basal forebrain cholinergic neurons (BFCNs), which project to these regions, play a critical role in regulating cognitive processes [25,26]. Notably, BFCN selective degeneration, a hallmark of Alzheimer’s disease (AD), triggers neurodegeneration in the hippocampus and frontal cortex, inducing cognition disruption [25,27]. Thus, PFOS may trigger memory and learning deficits by selectively damaging BFCNs, subsequently inducing secondary neurodegeneration in these key brain areas.

Supporting this hypothesis, our previous in vitro study demonstrated that PFOS exposure (24 h and 14 days) promotes BFCN cell death. This effect was partially mediated by disruption in cholinergic and glutamatergic transmission, along with overexpression of the acetylcholinesterase-S variant, driven partially by thyroid hormone (TH) signaling interference through a reduction in thyroid receptor α activity and an increase in triiodothyronine (T3) metabolism via the upregulation of deiodinase 3 [28]. THs support BFCN life maintenance and cognitive function, and their reduction triggers BFCN loss and cognitive decline [28]. However, these findings suggest that additional mechanisms are likely to contribute to PFOS neurotoxicity.

The neurotrophins are a group of secreted proteins that include nerve growth factor (NGF), brain-derived neurotrophic factor (BDNF), neurotrophin-3 (NT-3), and neurotrophin-4/5 (NT-4/5). NGF is abundantly expressed in BFCNs and plays a crucial role in their survival, synaptic plasticity, and cognitive function [29,30]. NGF is synthesized as preproNGF, which undergoes sequential processing to proNGF and finally to mature NGF (mNGF). This maturation primarily depends on plasmin, generated through the action of tissue plasminogen activator (tPA) or urokinase plasminogen activator (uPA), both negatively regulated by plasminogen activator inhibitor-1 (PAI-1) and neuroserpin [29,30]. Conversely, mNGF degradation is mediated by matrix metalloproteinases (MMPs), particularly MMP-9, and regulated by tissue inhibitor of metalloproteinases-1 (TIMP-1) [29,30].

The mNGF/tropomyosin receptor kinase A (TrkA) signaling pathway is essential for BFCN viability and function, while proNGF, acting through the P75^NTR^ receptor, exerts opposing effects [31]. The balance between mNGF and proNGF, along with TrkA/P75^NTR^ interactions, determines whether NGF signaling promotes neuronal survival or degeneration [29,30]. NGF deficiency, produced in Alzheimer’s disease (AD), contributes to BFCN loss and cognitive decline [30].

PFOS developmental exposure disrupts NGF dynamics in a context-dependent manner. In this sense, it reduces *NGF* expression in zebrafish larvae [32], increases *NGF* mRNA levels but decreases NGF protein in rat hippocampal neurons on postnatal day 35 (PND35) [33], and elevates *NGF* gene expression in the mouse brain cortex on PND35 [34]. PFOS also modulates NGF-processing enzymes. PFOS was shown to upregulate uPA in mouse hippocampal neurons [35], increase PAI-1 in rat cardiac cells [36], and MMP-9 in mouse testes and primary mouse Sertoli cells [37]. These data suggest that PFOS may induce BFCN neurodegeneration and impair cognitive function by dysregulating the proNGF/mNGF balance, potentially through altered processing enzyme activity.

Prostaglandin E2 (PGE2) plays a well-documented role in neurodegeneration, particularly in BFCN, where its overproduction has been linked to neuronal cell death and cognitive impairment [38,39,40,41]. Additionally, PGE2 has been shown to promote oxidative stress, further exacerbating neuronal damage [42,43]. Repeated PFOS exposure has been reported to increase the PGE2 levels in mouse liver [15] and in hepatocarcinoma cell lines [16]. A single PFOS treatment increases the levels of cyclooxygenase-2 (COX-2), an inducible enzyme, in human oral keratinocytes [17] and after repeated treatment in rat jejunal homogenates [18]. COX-2 works in concert with the constitutively expressed COX-1 to convert arachidonic acid into prostaglandin H2 (PGH2), the precursor for thromboxanes and prostaglandins [44,45]. PGH2 is subsequently converted to PGE2 by prostaglandin E synthase 1 (PTGES1) [44,45]. Therefore, PFOS-induced overexpression of COX-2 and/or PTGES1 may drive excessive PGE2 production, contributing to the observed neuronal loss and cognitive dysfunction.

Acute PFOS exposure induces oxidative damage across multiple experimental models. Single exposure in zebrafish embryos triggered the production of reactive oxygen species (ROS), lipid peroxidation, and upregulation of the NRF2/HO-1 pathway, along with the activation of antioxidant defenses (superoxide dismutase, catalase, glutathione peroxidase), ultimately leading to cell death [13]. Similar oxidative effects were observed in rat cerebellar granule cells (3 μM, single exposure) [14] and human SH-SY5Y neuroblastoma cells (50 μM, repeated exposure) [2], where N-acetylcysteine (NAC) treatment prevented PFOS-induced cytotoxicity, confirming oxidative stress mediation.

Notably, oxidative stress represents a well-established mechanism in neurodegenerative diseases, particularly in BFCN degeneration associated with cognitive impairment [19,46]. Given PFOS’s demonstrated capacity to (1) generate ROS, (2) disrupt redox homeostasis, and (3) activate compensatory antioxidant responses across diverse neural systems, these collective findings strongly suggest that PFOS may similarly induce BFCN degeneration through oxidative mechanisms, ultimately contributing to cognitive dysfunction.

THs have been shown to upregulate *NGF* gene expression in mouse L cells [47]. Experimental hypothyroidism was found to increase proNGF and P75^NTR^ levels while decreasing TrkA levels in the developing rat cerebral cortex [20]. In T3-L1 adipocytes, TH treatment elevated *PAI-1* gene expression, though this effect was not observed in vivo [48]. Clinical studies demonstrate that hyperthyroidism increases both uPA and PAI-1 levels in human plasma [49], while subclinical hypothyroidism specifically elevates PAI-1 [50]. THs have also been shown to increase the MMP-9 levels in primary bone marrow cells [51]. These collective findings indicate that THs play a regulatory role in the NGF/TrkA signaling pathway. Furthermore, THs modulate the NRF2 pathway in BFCNs [46]. Hypothyroid conditions were also associated with increased COX-2 expression in rat hippocampus and dentate gyrus [52] while decreasing PGE2 levels in rat uterine tissue [53]. Given these mechanisms, PFOS exposure may potentially induce oxidative stress, disrupt PGE2 and NGF/TrkA signaling pathways, and ultimately trigger BFCN degeneration through interference with TH activity, potentially contributing to cognitive dysfunction.

Based on the accumulated evidence presented above, we hypothesize that both acute and repeated PFOS exposure may disrupt TH action, subsequently inducing: (1) oxidative stress, (2) dysregulation of PGE2 signaling, and (3) impairment of NGF/TrkA pathways, ultimately leading to BFCN degeneration. To test this hypothesis, we conducted an in vitro experiment using SN56 cells (a BFCN model) exposed to PFOS (0.1–40 μM) and with siRNA against *PAI-1*, *MMP-9*, and/or *P75^NTR^* with or without the recombinant NGF (rNGF; 2 nM), with or without the recombinant uPA (ruPA; 15 nM), with or without MF-63 (PTGES-1 inhibitor; 1 μM), and with or without NAC (1 mM) to elucidate the molecular mechanisms underlying PFOS-induced BFCN neurodegeneration and identify potential therapeutic strategies to prevent or mitigate these effects associated with PFOS exposure.

## 2. Materials and Methods

### 2.1. Reagents

Perfluorooctane sulfonate (≥99%), dimethyl sulfoxide (DMSO), dithionitrobenzoic acid, dibutyryl-cAMP, MF-63, NAC, poly-L-lysine, rNGF, retinoic acid, 3-(4,5-dimethylthiazol-2-yl)-2,5-diphenyltetrazolium bromide (MTT), T3, and uPA were obtained from Sigma-Aldrich (Madrid, Spain). All other chemicals were of reagent grade with the highest available laboratory purity.

### 2.2. Culture Conditions

The SN56 cell line, derived from mouse cholinergic septal neurons [54], served as an experimental model of BFCNs to investigate the toxic effects of PFOS on this neuronal population and the underlying mechanisms. Cells were maintained in Dulbecco’s modified Eagle’s medium (DMEM; Sigma-Aldrich, Madrid, Spain) supplemented with penicillin/streptomycin, 10% fetal bovine serum (FBS), 2 mM L-glutamine, and 1 mM sodium pyruvate (Sigma-Aldrich, Madrid, Spain). Cultures were incubated at 37 °C under 5% CO_2_, and the medium was replaced every 48 h [55]. To induce morphological maturation and elevate choline acetyltransferase activity and acetylcholine levels by 3- to 4-fold, cells were differentiated by culturing for 72 h with 1 mM dibutyryl-cAMP and 1 µM retinoic acid (Sigma-Aldrich, Madrid, Spain) [56,57]. This differentiation step is critical, as neurotoxic xenobiotics exhibit greater effects on cholinergic pathways in differentiated cells [56,57]. Mycoplasma contamination was routinely assessed and excluded using the LookOut^®^ Mycoplasma Detection Kit (Sigma-Aldrich, Madrid, Spain).

To investigate the neurotoxic mechanisms of PFOS, SN56 cells were seeded at 2 × 10^6^ or 1 × 10^6^ (1- or 14-day treatment, respectively) cells/well in 6-well plates and subjected to comprehensive analysis. We quantified: (1) cellular content of hydrogen peroxide (H_2_O_2_), malondialdehyde (MDA), protein carbonyls, and PGE2, uPA, PAI-1, MMP-9, P75^NTR^, TrkA, proNGF, mNGF, COX-2, NRF2, superoxide dismutase 1 (SOD-1), heme oxygenase 1 (HO-1), and PTGES-1; (2) gene expression of *NGF*, *PAI-1*, *MMP-9*, and *P75^NTR^*; and (3) consequences following RNAi-mediated knockdown *PAI-1*, *MMP-9*, and *P75^NTR^*.

Treatments tested our central hypothesis through the daily administration of PFOS (0.1–40 μM) for 1 or 14 days, with parallel combinatorial interventions: MF-63 (1 μM), a selective PTGES-1 inhibitor, T_3_ (15 nM), NAC (1 mM), ruPA (15 nM; MBS638074, MyBioSource, CA, USA), and/or rNGF (2 nM; MBS7115005, MyBioSource, San Diego, CA, USA). PFOS stock aliquots were progressively diluted in sterile culture medium to generate working concentrations, ensuring that the final treatment solutions contained 0.1% DMSO as the carrier solvent. The choice of T3 was based on: (1) our previous study that showed that PFOS alters THs signaling in cholinergic neurons [29]; (2) evidence showing that THs regulate multiple neuronal survival pathways including NGF expression, antioxidant activity via NRF2, and modulation of inflammation [20,46,47,51]; and (3) the fact that T3 is the biologically active form of THs, capable of directly activating nuclear TH receptors and restoring downstream signaling. All experimental conditions included matched vehicle controls, and the vehicle-only negative control was replicated in ≥3 independent wells per treatment group. As no statistically significant differences were observed between the control groups at the 1- and 14-day timepoints, the data were combined and displayed as a unified control (white bar).

PFOS exhibits rapid systemic distribution with significant bioaccumulation potential, particularly in neural tissues [58] and shows prolonged persistence in humans (serum half-life: 5.4 years; [5]). Human biomonitoring studies report plasma concentrations ranging from 0.002 to 0.23 μM in the general population and from 0.988 to 10 μM in occupationally exposed individuals [59]. For our experimental design, we selected a concentration range of 0.1–40 μM PFOS, encompassing both environmentally relevant exposures and concentrations previously shown to elicit toxic mechanisms in vitro [2,3,60]. The 10 μM concentration was specifically chosen for mechanistic studies based on its established ability to: (1) compromise cellular viability, (2) induce oxidative stress, and (3) disrupt both the prostaglandin and NGF/TrkA/P75^NTR^ signaling pathways in SN56 cells following 1- or 14-day exposures.

### 2.3. Prostaglandin E2 Content Assessment

Culture medium was collected and processed for PGE2 quantification using a commercial ELISA kit (ab133021; Abcam, Cambridge, UK) according to the manufacturer’s instructions. Absorbance measurements were performed at 405 nm using a Fluoroskan FL microplate reader (Thermo Fisher Scientific, Madrid, Spain). PGE2 concentrations (pg/mL) were normalized to total intracellular protein content (μg/mL), with final values expressed as pg PGE2/μg protein.

### 2.4. Oxidative Stress Assessment

To evaluate oxidative stress parameters, we quantified protein carbonylation, H_2_O_2_ levels, and MDA formation as markers of protein oxidation, ROS generation, and lipid peroxidation, respectively. These analyses were performed using commercially available Abcam assays: Lipid Peroxidation MDA Assay Kit (ab233471; Cambridge, UK), Hydrogen Peroxide Assay Kit (ab102500), and Protein Carbonyl Content Assay (ab126287), according to the manufacturer’s protocols.

For each oxidative stress marker, cell pellets containing 1 × 10^6^ cells were homogenized in ice-cold lysis buffers provided with the respective kits. Processed samples and appropriate standards were then aliquoted into 96-well plates, following assay-specific requirements. Spectrophotometric measurements were conducted using a Thermo Fisher Fluoroskan FL microplate reader (Madrid, Spain), with absorbance readings performed at distinct wavelengths for each analyte: 370 nm for protein carbonyls, 572 nm for H_2_O_2_, and 532 nm for MDA quantification.

The oxidative stress biomarkers were quantified using standard curves generated for each assay, with results expressed in standardized units: H_2_O_2_ concentrations as nmol/mL. At the same time, protein carbonyl and MDA levels were normalized to total protein content and reported as nmol/mg protein. This comprehensive approach allowed for the simultaneous evaluation of multiple oxidative damage pathways under consistent experimental conditions, ensuring comparability across biomarkers.

### 2.5. Quantification of Target Proteins

Following PBS washing (pre-chilled), cells were mechanically detached and lysed in RIPA buffer (Thermo Scientific, Madrid, Spain) supplemented with a protease inhibitor cocktail. The lysates were centrifuged at 10,000× *g* for 10 min at 4 °C, removing cellular debris. Clarified supernatant was carefully aspirated for downstream studies. Protein concentration was determined using a BCA kit (Thermo Fisher Scientific, Madrid, Spain).

The protein levels of COX-2, PTGES-1, MMP9, PAI-1, uPA, NRF2, SOD-1, HO-1, TrkA, P75^NTR^, proNGF, and mNGF were quantified using commercially available ELISA kits (MBS3806086, MBS726011, MBS702680, MBS261751, MBS044609, MBS776676, MBS451661, MBS267777, MBS761054, MBS269783, MBS706350, and MBS175827, respectively; MyBioSource, San Diego, CA, USA), strictly adhering to the producer’s protocols. To ensure specificity, negative controls were included for each target protein. Data normalization was determined using cellular protein content and presented as nanograms of target protein per milligram of total protein (ng/mg).

### 2.6. Gene Expression Measurement

Total RNA was isolated and complementary DNA (cDNA) synthesized using established methodologies [61]. Gene expression profiling was performed using validated primer sets (SuperArray Bioscience, Frederick, MD, USA) targeting key transcripts: *P75^NTR^* (PPM04327F), *NGF* (PPM03596B), *MMP9* (PPM03661C), *PAI-1* (PPM03093C), and the reference genes *β-actin* (PPM02945B) and glyceraldehyde-3-phosphate dehydrogenase (PPM02946E). The qPCR analysis followed the MIQUE requirements.

Quantitative PCR amplification was carried out in a CFX96 thermocycler (Bio-Rad, Madrid, Spain) using SYBR Green Master Mix (PA-012; SuperArray Bioscience, Frederick, MD, USA). The thermal profile consisted of an initial denaturation at 95 °C for 10 min, followed by 40 cycles of denaturation (95 °C, 15 s) and annealing/extension (72 °C, 30 s). All reactions were performed in technical triplicates with appropriate negative controls. Normalization of expression data was performed against *β-actin* and glyceraldehyde-3-phosphate dehydrogenase as reference genes, and the data were analyzed using the comparative Ct method (2^−ΔΔCt^). Relative quantification of transcript levels was calculated as fold-change values relative to control conditions, following established normalization procedures [62].

### 2.7. siRNA Transfection and Gene Silencing Validation

Cells were seeded at a density of 1 × 10^6^ cells per well and transfected using the HiPerfect Transfection Reagent (Qiagen, Barcelona, Spain). siRNA duplexes were designed using the HiPerformance Design Algorithm (Novartis AG, Basel, Switzerland) and obtained from Qiagen, targeting the following murine genes: *P75^NTR^* (GS18053), *MMP9* (GS17395), and *PAI-1* (GS319433). Silencing controls were carried out using the AllStars Negative Control siRNA (Qiagen, Barcelona, Spain).

At 48 h post-transfection, silencing efficiency was assessed via RT-PCR using gene-specific primers for target genes (Qiagen, Barcelona, Spain). To determine whether gene knockdown affected cellular viability, an MTT assay was conducted. Following a 24 h incubation with siRNA, cells were washed with PBS and subsequently treated with PFOS or the control medium for either 1 or 14 days.

### 2.8. Cell Viability Determination (Caspases 3/7 and MTT Assays)

Cell viability following PFOS treatment was evaluated using the MTT assay, as previously described [63]. To determine whether PFOS exposure triggered apoptotic pathways, caspase-3/7 activity was measured using the Caspase-Glo^®^ 3/7 luminescence assay (Promega, Madrid, Spain), in accordance with the manufacturer’s protocol.

### 2.9. Statistical Analysis

All experimental conditions were assessed in triplicate, with three independent biological replicates per condition (*n* = 9), ensuring robust and reproducible results. Data are presented as mean values ± standard error of the mean (SEM). For comparisons between treatment groups and controls, statistical significance was determined using an unpaired two-tailed Student’s *t*-test (Section 3.1).

To evaluate the interaction between gene manipulation and treatment effects, a two-way analysis of variance (ANOVA) was applied, whereas a one-way ANOVA was employed to assess the concentration-dependent impact of PFOS concentrations on cellular responses. Post hoc multiple comparisons were conducted using Tukey’s test, with a significance threshold set at *p* ≤ 0.05. All statistical analyses were performed using GraphPad Prism 5.1 (GraphPad, Boston, MA, USA).

## 3. Results

### 3.1. Gene Knockdown Analysis

**Figure 1:**
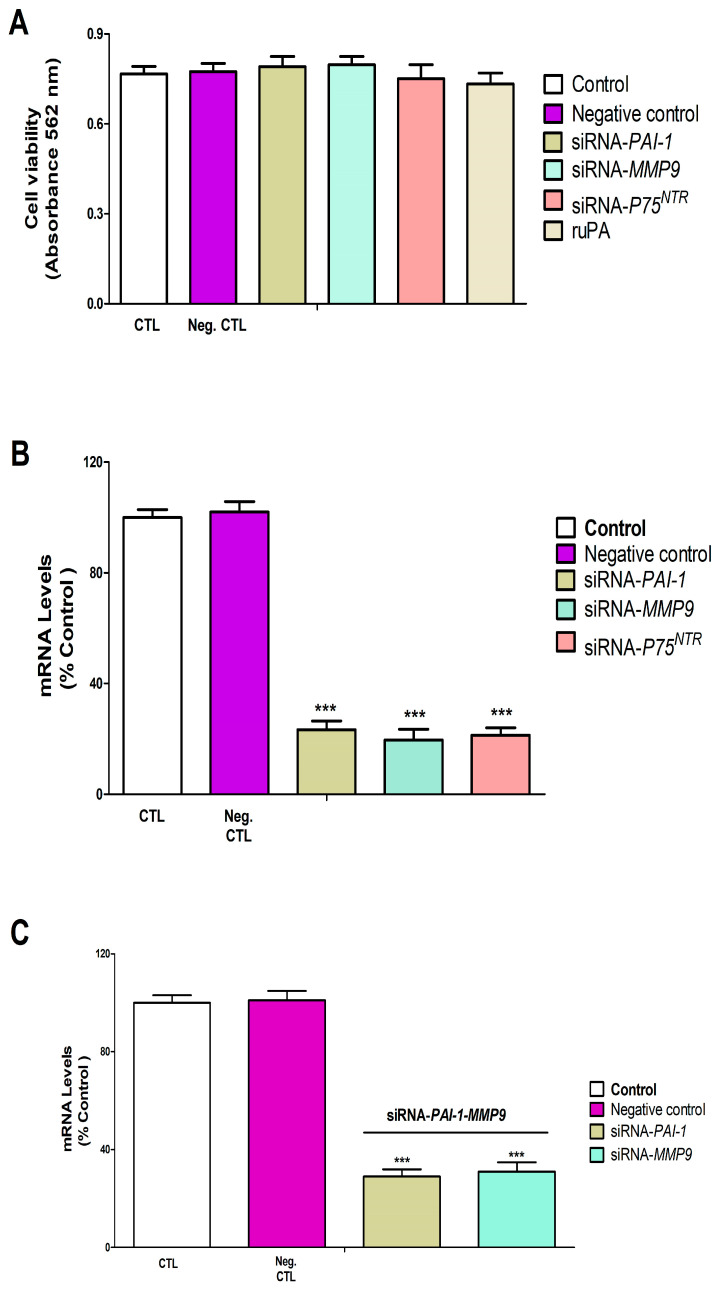
*PAI-1*, *MMP-9*, and *P75^NTR^* silencing effect on SN56 cell viability and gene expression. Control: SN56 cells transfected without siRNA. Negative (Neg.) control: SN56 cells transfected with control siRNA. PAI-1-siRNA: transfected with siRNA against *PAI-1*. MMP9-siRNA: transfected with siRNA against *MMP-9*. P75^NTR^-siRNA: transfected with siRNA against *P75^NTR^*. MTT analysis shows that *PAI-1*, *MMP-9*, and *P75^NTR^* knockout did not significantly induce cell damage after 48 h (**A**). *PAI-1*, *MMP-9*, and *P75^NTR^* downregulation could be detected by RT-PCR analysis 48 h after transfection (**B**,**C**). Values are given as mean ± SEM of three separate experiments from cells of different cultures, each one performed in triplicate. *** *p* ≤ 0.001 compared to the control.

To evaluate the functional role of PAI-1, MMP-9, and P75^NTR^ in PFOS neurotoxicity, we first validated the efficiency and specificity of their silencing using siRNA. Our experiments with SN56 cells showed that transfection with siRNA targeting individual genes (*PAI-1*, *MMP-9*, or *P75^NTR^*) or combined siRNAs (*PAI-1* and *MMP-9* together) did not compromise cell viability compared to the control siRNA transfection (Figure 1A). The control siRNA transfection, as expected, did not affect the expression levels of *PAI-1*, *MMP-9*, or *P75^NTR^* genes (Figure 1B,C). However, when we specifically targeted these genes with their respective siRNAs, we observed successful knockdown. Single siRNA transfections effectively reduced the expression of their target genes, and the dual *PAI-1*/*MMP-9* siRNA combination simultaneously decreased the expression of both genes (Figure 1B,C). Importantly, the silencing efficiency was maintained whether the siRNAs were delivered individually or in combination. The successful validation of specific silencing without affecting cell viability allowed us to use these models in subsequent experiments to unravel causal mechanisms.

### 3.2. Assessment of uPA, PAI-1, and MMP-9 Protein Content

To determine whether PFOS alters the plasmin/urokinase system, which is crucial for NGF maturation, we investigated its effects on the uPA, PAI-1, and MMP-9 levels. Plasmin, generated from plasminogen by the action of uPA (and negatively regulated by PAI-1), converts proNGF into mature mNGF, while MMP-9 degrades mNGF. We therefore hypothesized that PFOS would disrupt this balance.

**Figure 2:**
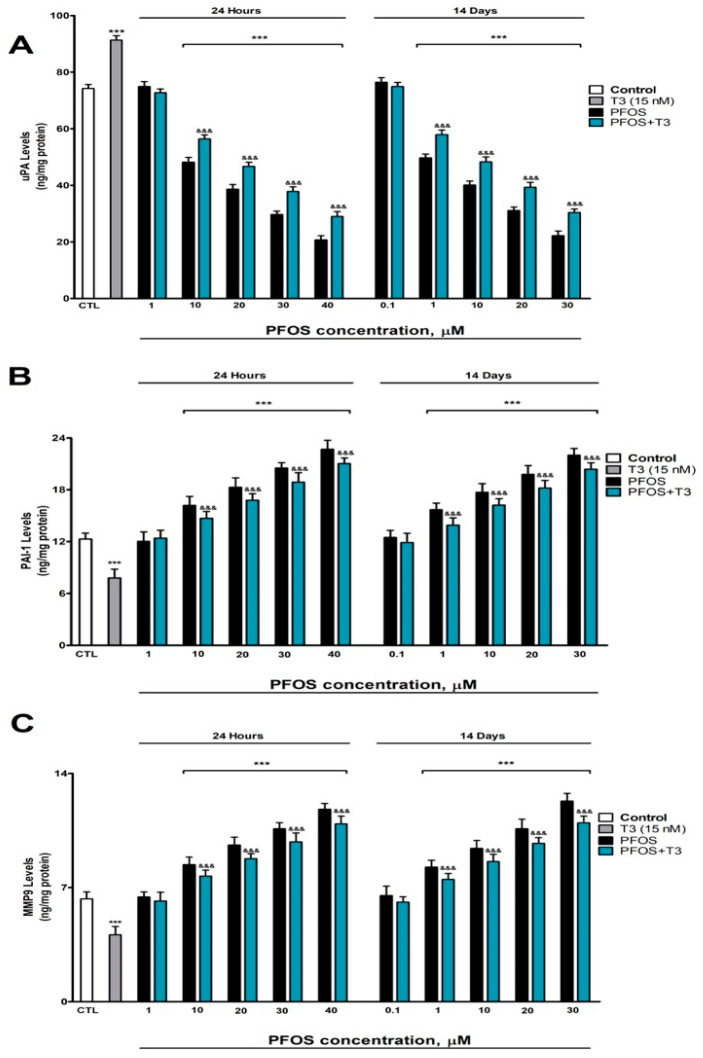
PFOS (0.1–40 µM) effects on (**A**) uPA, (**B**) PAI-1, and (**C**) MMP-9 levels in SN56 cell homogenates after one- and fourteen-days of treatment. The mean ± SEM was obtained from data of three replicates of cultures performed three different times. *** *p* ≤ 0.001, significantly different from the controls. ^&&&^ *p* ≤ 0.001 compared to the PFOS treatment.

The protein levels of MMP-9, uPA, and PAI-1 were analyzed in SN56 cells after exposure to PFOS at concentrations ranging from 0.1 µM to 40 µM for 1 and 14 days. PFOS treatment significantly reduced the uPA levels (Figure 2A) while increasing PAI-1 (Figure 2B) and MMP-9 (Figure 2C) compared to the controls. These effects were observed after just one day of exposure, starting at a concentration of 10 µM, and became more pronounced with longer treatment (fourteen days), where changes were detectable at 1 µM and intensified with higher concentrations (Figure 2). These effects at 14 days appeared at lower concentrations (≥1 µM) than at 24 h (≥10 µM), reflecting the accumulation and progressive toxicity of PFOS. Additionally, T3 treatment alone significantly elevated the uPA levels (Figure 2A) while reducing PAI-1 (Figure 1B) and MMP-9 (Figure 2C). When T3 was co-administered with PFOS, it partially counteracted the alterations induced by PFOS alone, mitigating its impact on these proteins (Figure 2).

These results indicate that PFOS perturbs the NGF processing system by reducing uPA, increasing PAI-1, and elevating MMP-9, which would favor proNGF accumulation and mNGF degradation. Importantly, the effects at 14 days appeared at lower concentrations (≥1 µM) than at 24 h (≥10 µM), indicating a time-dependent effect of exposure.

### 3.3. Analysis of P75^NTR^ and TrkA Protein Content and NGF Gene Expression

**Figure 3:**
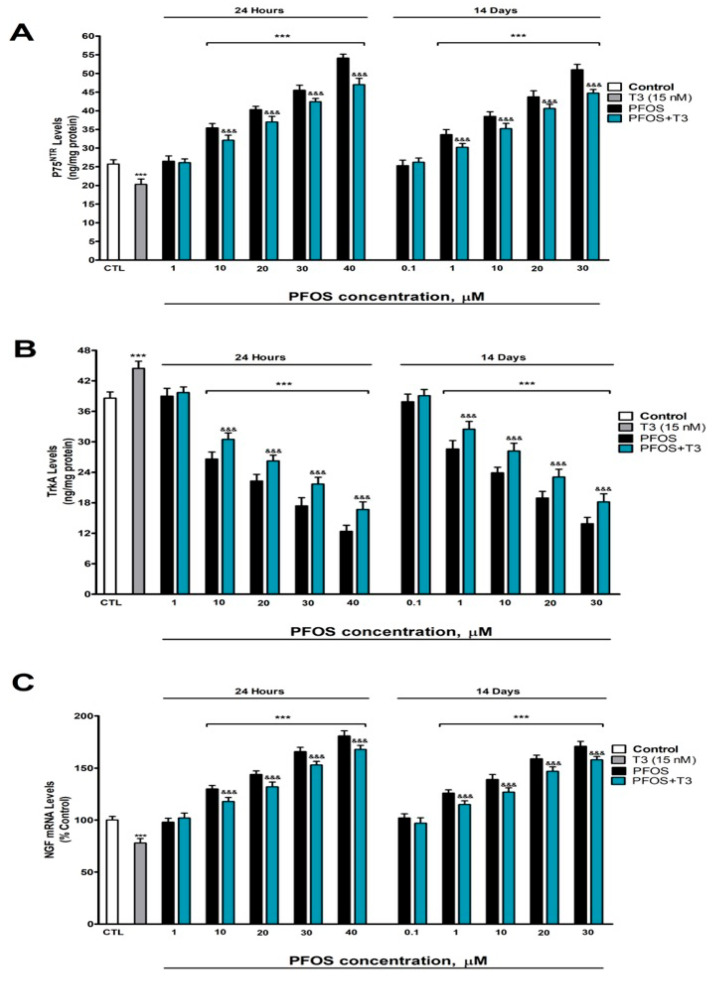
PFOS (0.1–40 µM) effects on (**A**) P75^NTR^, and (**B**) TrkA levels, and (**C**) *NFG* gene expression in SN56 cell homogenates after one- and fourteen-days of treatment. Data represents the mean ± SEM of three separate experiments from cells of different cultures, each one performed in triplicate. *** *p* ≤ 0.001 compared to the control. ^&&&^ *p* ≤ 0.001 compared to the PFOS treatment.

Given that the balance between TrkA-mediated pro-survival signaling and P75^NTR^-mediated pro-apoptotic signaling determines the fate of cholinergic neurons, we assessed whether PFOS alters this equilibrium. PFOS treatment induced a concentration-dependent elevation in P75^NTR^ levels and in *NGF* expression, as well as a reduction in TrkA levels compared to the control group, with statistically significant effects observed at concentrations ≥10 µM after 1 day and ≥1 µM after 14 days (Figure 3A–C). These effects at 14 days appeared at lower concentrations (≥1 µM) than at 24 h (≥10 µM), reflecting the accumulation and progressive toxicity of PFOS. In contrast, treatment with T3 alone reduced the P75^NTR^ protein levels (Figure 3A) and *NGF* gene expression (Figure 3C) while increasing TrkA content (Figure 3B). When T3 was co-administered with PFOS, it partially counteracted the alterations induced by PFOS alone, mitigating its effects on these markers (Figure 3). Taken together, these data show that PFOS shifts the neurotrophic balance toward a pro-apoptotic signaling profile, increasing P75^NTR^ and reducing TrkA, which could directly contribute to the observed neuronal death.

### 3.4. Analysis of proNGF and mNGF Protein Content

To confirm whether changes in processing enzymes (uPA, PAI-1, MMP-9) translate into alterations in NGF isoforms, we quantified the levels of proNGF (the pro-apoptotic form) and mNGF (the neuroprotective mature form).

**Figure 4:**
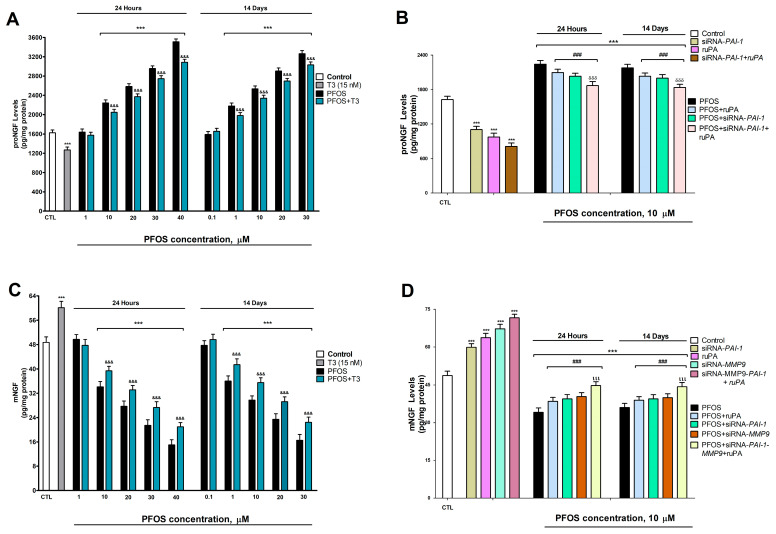
PFOS (0.1–40 µM) effects on (**A**) proNGF and (**C**) mNGF protein content after one- and fourteen-days of treatment. Effects of *PAI-1* knockdown, treatment with PFOS (10 µM) or ruPA (15 µM), ruPA treatment of *PAI-1* silenced cells or PFOS co-treatment with ruPA of wildtype cells or PFOS co-treatment with ruPA of *PAI-1* silenced cells on proNGF protein content (**B**). Effects of *PAI-1* or *MMP-9* knockdown, treatment with PFOS (10 µM) or ruPA (15 µM), ruPA treatment of simultaneous *PAI-1* and *MMP-9* silenced cells, or PFOS co-treatment with ruPA of wildtype cells or PFOS co-treatment with ruPA of single or simultaneous *PAI-1* and/or *MMP-9* silenced cells on mNGF protein content (**D**). Data represent the mean ± SEM of three separate experiments from cells of different cultures, each one performed in triplicate. *** *p* < 0.001 compared to the control. ^###^ *p* ≤ 0.001 compared to the PFOS treatment. ^&&&^ *p* ≤ 0.001 compared to the PFOS treatment. ^δδδ^ *p* ≤ 0.001 compared to the ruPA co-treated cells with PFOS. ^ιιι^ *p* ≤0.001 compared to the PFOS treatment of *MMP-9-silenced* cells.

In SN56 cells treated with PFOS, we observed an increase in proNGF protein levels (Figure 4A) alongside a significant decrease in mNGF protein content (Figure 4C). These effects were evident after just one day of exposure (starting at 10 µM PFOS) and following fourteen days of treatment (with changes detectable at 1 µM). The impact of PFOS intensified with higher concentrations (Figure 4A,C). These effects at 14 days appeared at lower concentrations (≥1 µM) than at 24 h (≥10 µM), reflecting the accumulation and progressive toxicity of PFOS.

In contrast, when wild-type cells were subjected to *PAI-1* silencing, T3 treatment, or ruPA administration alone, the proNGF levels decreased (Figure 4A,C) while the mNGF levels increased, a trend also seen with *MMP-9* silencing (Figure 4D). Combined PFOS treatment with T3 incompletely mitigated the effects of PFOS alone, reducing its influence on these markers (Figure 4).

Additionally, combining PFOS treatment with ruPA or applying PFOS to *PAI-1*-silenced cells partially counteracted the rise in proNGF (Figure 3B). Similarly, the decrease in mNGF was partially attenuated when PFOS was administered alongside ruPA or in *PAI-1*- or *MMP-9*-silenced cells (Figure 4D). The most substantial reduction in proNGF elevation occurred when PFOS and ruPA were co-administered in *PAI-1*-silenced cells (Figure 4C). Likewise, the strongest attenuation of mNGF loss was observed in cells with simultaneous *PAI-1* and *MMP-9* silencing and co-treated with PFOS and ruPA (Figure 4D). However, even under these conditions, the effects of PFOS were not completely reversed.

These findings demonstrate that PFOS alters NGF processing toward a pro-degenerative profile, and that interventions targeting the plasmin system (ruPA) or its inhibitors (PAI-1/MMP-9 silencing) partially attenuate these effects.

### 3.5. NRF2 Pathway Assessment (HO-1, SOD-1, and NRF2 Protein Levels Quantification)

To assess whether PFOS compromises cellular antioxidant defenses, we analyzed the NRF2 pathway, a master regulator of the antioxidant response that controls the expression of enzymes such as SOD-1 and HO-1.

**Figure 5:**
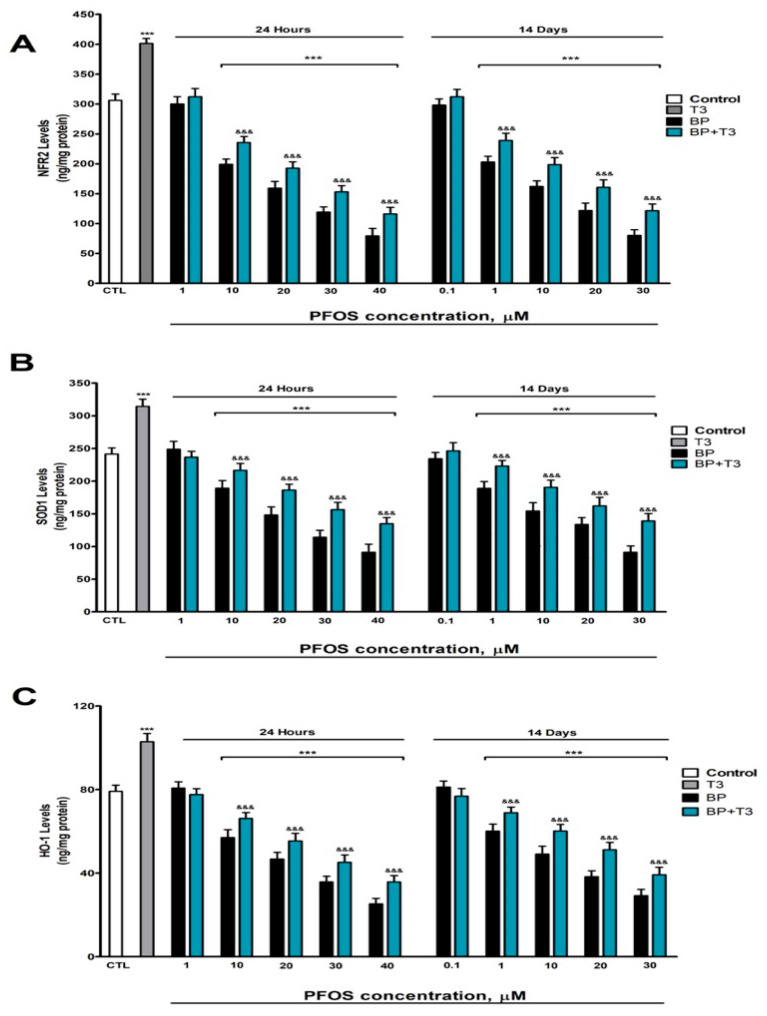
PFOS (0.1–40 µM) effects on (**A**) NRF2, (**B**) SOD-1, and (**C**) HO-1 contents in SN56 cell homogenates after one- and fourteen-days of treatment. Data represent the mean ± SEM of three separate experiments from cells of different cultures, each one performed in triplicate. *** *p* ≤ 0.001 compared to the control. ^&&&^ *p* ≤ 0.001 compared to the PFOS treatment.

A concentration-dependent decrease in key antioxidant protein levels, including NRF2 (Figure 5A), SOD-1 (Figure 5B), and HO-1 (Figure 5C) was observed. These reductions were detectable after just one day of treatment at concentrations of 10 µM or higher, and following prolonged exposure (14 days), where effects were evident at concentrations as low as 1 µM. These effects at 14 days appeared at lower concentrations (≥1 µM) than at 24 h (≥10 µM), reflecting the accumulation and progressive toxicity of PFOS.

In contrast, T3 treatment alone showed a protective effect by significantly boosting the cellular levels of NRF2 (Figure 5A), SOD-1 (Figure 5B), and HO-1 (Figure 5C). Combined PFOS treatment with T3 partially, but significantly, mitigated PFOS’s detrimental effects, helping to maintain higher levels of these antioxidant proteins compared to cells treated with PFOS alone (Figure 5).

The suppression of the NRF2 pathway by PFOS, together with the reduction in SOD-1 and HO-1, indicates compromised cellular antioxidant capacity, which would favor the accumulation of oxidative damage.

### 3.6. Analysis of Oxidative Stress

**Figure 6:**
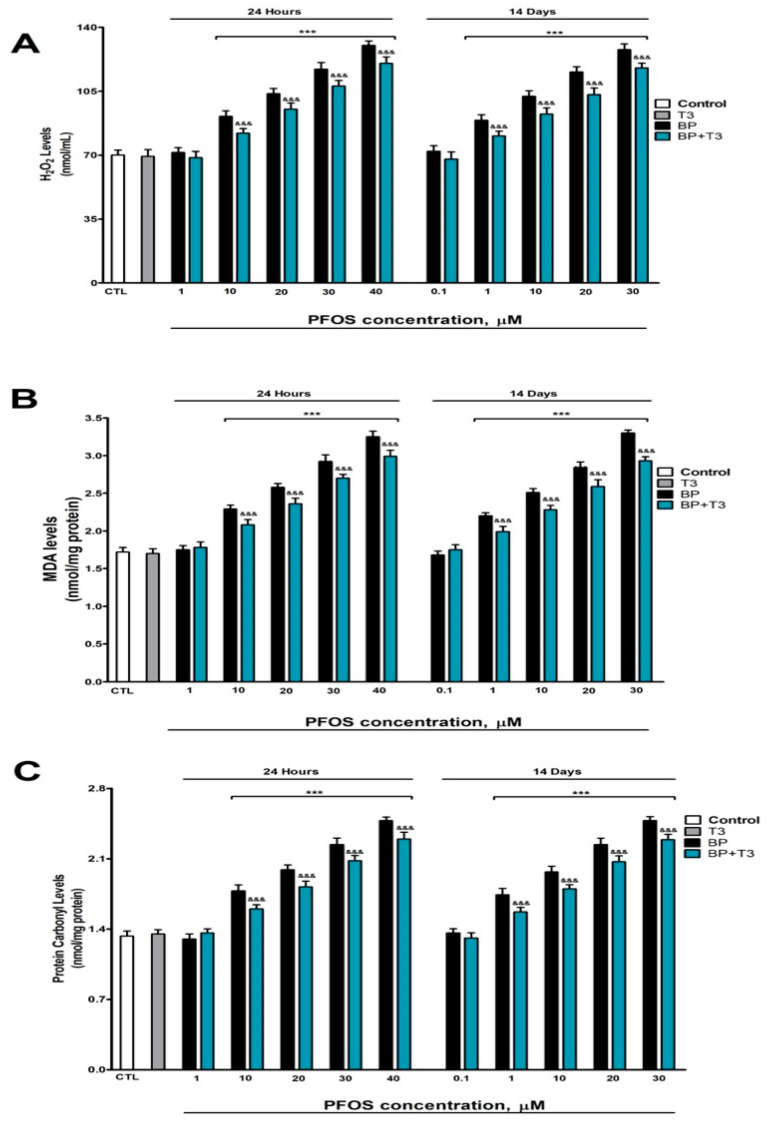
PFOS (0.1–40 µM) effects on (**A**) H_2_0_2_, (**B**) MDA, and (**C**) protein carbonyl contents in SN56 cell homogenates after one- and fourteen-days of treatment. Data represents the mean ± SEM of three separate experiments from cells of different cultures, each one performed in triplicate. *** *p* ≤ 0.001 compared to the control. ^&&&^ *p* ≤ 0.001 compared to the PFOS treatment.

Since oxidative stress is a central mechanism in neurodegeneration, we quantified direct markers of oxidative damage: H_2_O_2_ (reactive oxygen species), MDA (lipid peroxidation), and protein carbonyls (protein oxidation). Both the one-day (≥10 µM) and fourteen-day (≥1 µM) treatments resulted in elevated levels of H_2_O_2_, MDA, and protein carbonyls, with these effects showing a clear concentration-dependent enhancement (Figure 6). These effects at 14 days appeared at lower concentrations (≥1 µM) than at 24 h (≥10 µM), reflecting the accumulation and progressive toxicity of PFOS. In contrast, T3 treatment by itself did not significantly alter the basal levels of H_2_O_2_ (Figure 6A), MDA (Figure 6B), or protein carbonyls (Figure 6C). Combined PFOS treatment with T3 partially mitigated the oxidative stress caused by PFOS alone. This partial protective effect was observed across all markers measured (Figure 6).

The concentration- and time-dependent increase in all oxidative stress markers confirms that PFOS induces a sustained pro-oxidant state in cholinergic neurons. The complete protection by NAC validates the central role of oxidative stress in this toxicity.

### 3.7. Analysis of COX-2 and PGE2 Content

Since PGE2 (a pro-inflammatory lipid mediator) is synthesized from arachidonic acid via COX-2 and PTGES1, and its overproduction is associated with neurodegeneration, we investigated whether PFOS activates this inflammatory pathway.

**Figure 7:**
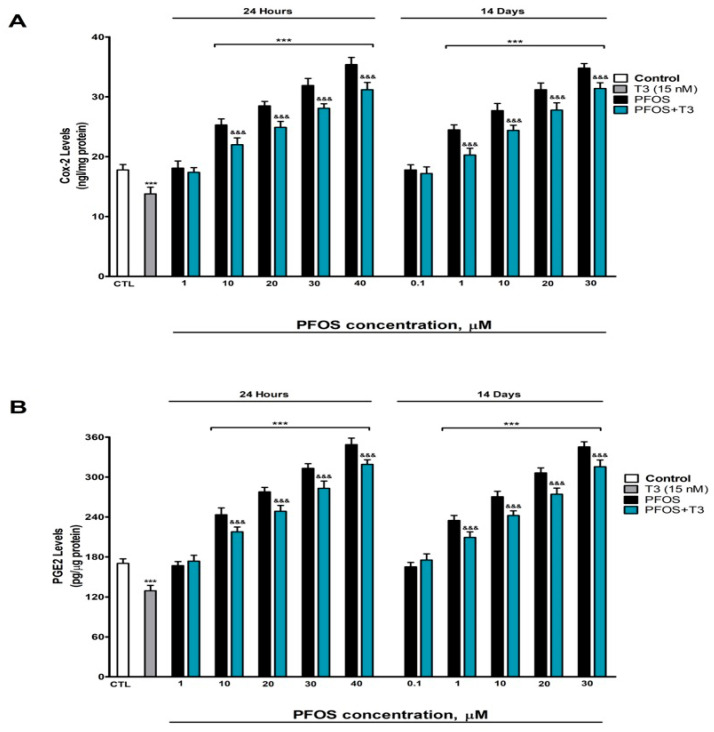
PFOS (0.1–40 µM) effects on (**A**) COX-2, and (**B**) PGE2 content in SN56 cell after one- and fourteen-days of treatment. Data represents the mean ± SEM of three separate experiments from cells of different cultures, each one performed in triplicate. *** *p* ≤ 0.001 compared to the control. ^&&&^ *p* ≤ 0.001 compared to the PFOS treatment.

The treatment resulted in a marked elevation of both the COX-2 (Figure 7A) and PGE2 (Figure 7B) levels compared to the untreated controls. Notably, these inflammatory effects appeared rapidly after just 24 h of exposure at concentrations as low as 10 µM and after prolonged exposure (14 days), responding to PFOS concentrations as low as 1 µM, with the inflammatory response intensifying in a concentration-dependent manner (Figure 7). These effects at 14 days appeared at lower concentrations (≥1 µM) than at 24 h (≥10 µM), reflecting the accumulation and progressive toxicity of PFOS. In contrast, T3 treatment exhibited anti-inflammatory properties on its own, significantly reducing the baseline levels of both COX-2 and PGE2 (Figure 7). When we combined T3 with PFOS treatment, we observed that T3 provided partial protection against PFOS-induced inflammation, effectively blunting but not completely preventing the upregulation of these inflammatory markers (Figure 7).

These results indicate that PFOS activates the COX-2/PGE2 pathway, which could contribute to the inflammatory environment that promotes neuronal death. T3 partially attenuates this activation, suggesting a link to thyroid disruption.

### 3.8. Cell Viability Assessment and Caspases 3/7 Activation Determination

To integrate the observed molecular alterations with a functional outcome, we assessed cell viability and caspase-3/7 activation (apoptosis markers) after PFOS exposure, and tested rescue strategies based on our mechanistic hypotheses.

**Figure 8:**
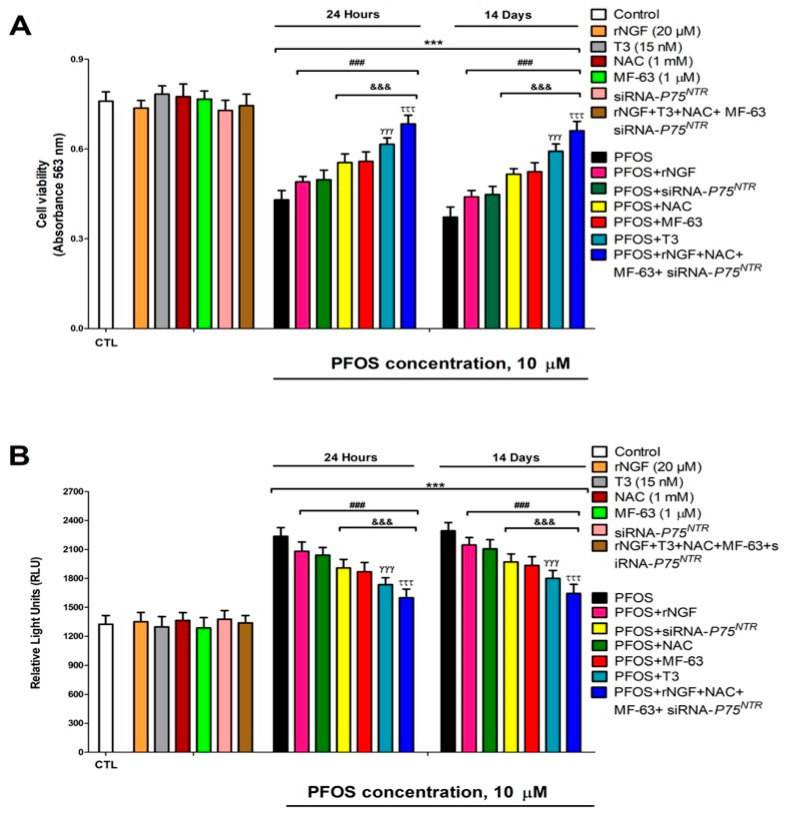
Analysis of cell viability (**A**) and caspases 3/7 activity (**B**) in PFOS (10 µM) wild-type or *P75^NTR^* silenced cells co-treated with or without T3 (15 nM), and/or rNGF, and/or NAC (1 µM). Cell viability was determined by the MTT test. Data represent the mean ± SEM of three separate experiments from cells of different cultures, each one performed in triplicate. *** *p* < 0.001 compared to the control. ^###^ *p* ≤ 0.001 compared to the PFOS treatment. ^&&&^ *p* ≤ 0.001 compared to the PFOS treatment of *P75^NTR^*-silenced cells. ^γγγ^ *p* ≤0.001 compared to the PFOS co-treatment with MF-63. ^τττ^ *p* ≤0.001 compared to the T3 co-treated cells with PFOS.

Our experiments revealed a concentration-dependent decrease in cell viability following PFOS exposure. After just one day of treatment, viability began declining at 10 µM PFOS, while fourteen-day exposure showed effects at concentrations as low as 1 µM. In both cases, the cytotoxic effects became more pronounced with increasing PFOS concentrations (Figure 8A). Interestingly, none of our protective treatments, including T3, rNGF, MF-63, or NAC administration in wild-type cells, *P75^NTR^* silencing alone, or combined treatments in *P75^NTR^*-silenced cells, showed any negative impact on cell viability under normal conditions (Figure 8B). However, when these interventions were applied alongside PFOS exposure, they demonstrated varying degrees of protective effects. All tested treatments, T3, rNGF, MF-63, or NAC co-treatment in wild-type cells and PFOS exposure in *P75^NTR^*-silenced cells, partially mitigated the viability reduction caused by PFOS alone (Figure 8A). Among these, NAC and MF-63 showed stronger protective effects than rNGF or *P75^NTR^* silencing. Notably, T3 outperformed both NAC and MF-63 in preserving cell viability during PFOS exposure. The most robust protection came from combining all four treatments (T3, rNGF, MF-63, and NAC) in *P75^NTR^*-silenced cells, which achieved the greatest attenuation of PFOS-induced viability loss (Figure 8A). However, even this comprehensive approach did not completely neutralize PFOS toxicity. Importantly, control experiments confirmed no significant differences between the vehicle-treated and untreated cells.

Our results also show that PFOS exposure triggered significant activation of caspases 3/7, key mediators of apoptosis. This activation showed clear concentration-dependent patterns, detecting effects after one day at 10 µM concentrations, while fourteen-day exposure produced measurable activation at just 1 µM, with progressively stronger effects at higher concentrations (Figure 8B). Importantly, none of our protective interventions, including individual treatments with T3, rNGF, MF-63, or NAC in wild-type cells, *P75^NTR^* silencing alone, or combined treatments in *P75^NTR^*-silenced cells, induced caspase activation on their own (Figure 8B). However, when administered alongside PFOS, these treatments showed varying degrees of effectiveness in reducing caspase activation. All protective strategies, whether using T3, rNGF, MF-63, or NAC in non-transfected cells or PFOS exposure in *P75^NTR^*-knockdown cells, successfully attenuated PFOS-induced caspase activation (Figure 8B). Among these, NAC and MF-63 showed superior protective effects compared to rNGF treatment or *P75^NTR^* silencing alone. Notably, T3 treatment showed even greater efficacy than either NAC or MF-63 in suppressing caspase activation. The most comprehensive protection was achieved through combined treatment with T3, rNGF, MF-63, and NAC in *P75^NTR^*-silenced cells, which produced the strongest reduction in caspase activation (Figure 8B). However, even this combined approach did not completely block PFOS-induced apoptosis. These caspase activation findings closely paralleled our cell viability data, strongly suggesting that PFOS induces cell death primarily by apoptotic pathways.

The cell viability reduction and caspases 3/7 activation at 14 days appeared at lower concentrations (≥1 µM) than at 24 h (≥10 µM), reflecting the accumulation and progressive toxicity of PFOS. PFOS-induced cell death is partially mitigated by interventions targeting multiple pathways. The most robust protection with combined treatment suggests that these pathways act in an additive/synergistic manner.

## 4. Discussion

PFOS treatment (1- and 14-day exposures) induced concentration-dependent increases (starting at 10 µM or 1 µM, respectively) in *NGF* expression and protein levels of proNGF, P75^NTR^, PAI-1, and MMP-9 while reducing the uPA, mNGF, and TrkA protein levels. Combining PFOS treatment with T3 partially mitigated the alteration of these targets. To our knowledge, this is the first study to investigate the effects of PFOS on TrkA, P75^NTR^, proNGF, and mNGF. Previous reports indicate that developmental PFOS exposure upregulates *NGF* expression and NGF protein levels in mice brain cortex at PND21 [34] but increases *NGF* gene expression while decreasing NGF protein in rat hippocampal neurons at PND35 [33], aligning with our findings. These opposing effects may stem from differences in the NGF isoforms detected (proNGF vs. mNGF), experimental models (in vivo vs. in vitro), species, brain regions, or developmental stages. Although PFOS effects on TrkA were previously unexplored, acute PFOS exposure reduces TrkB protein levels in SH-SY5Y cells [63], suggesting broader regulation of neurotrophin receptors by PFOS.

Similarly, PFOS upregulates uPA in mouse hippocampal neurons [34], though the discrepancy with our results may reflect differences in model systems, exposure duration, concentrations, or neuronal cell origins. Further supporting our data, PFOS elevates PAI-1 in rat cardiac cells [36] and MMP-9 in mouse primary Sertoli cells and testes following repeated exposure [37]. The observed increase in proNGF protein levels could be partially mediated by elevated *NGF* expression and altered uPA/PAI-1 levels, which regulate its conversion to mNGF [29,30]. It is interesting to note that the combination of ruPA and PAI-1 silencing did not show an additive or synergistic effect on proNGF reduction. One possible explanation is that the addition of exogenous ruPA may saturate the conversion of plasminogen to plasmin, even in the presence of PAI-1, rendering further PAI-1 reduction redundant. Alternatively, it is plausible that PFOS affects substrate (plasminogen) availability or the activity of other proteases, thus limiting the combined effect. Future studies that directly measure plasminogen levels and proteolytic activity, or that employ lower concentrations of ruPA, could clarify this interaction. While no prior studies have examined the effects of PFOS on tPA or neuroserpin, additional regulators of this process, these factors may also contribute. The reduction in mNGF levels seems to result partly from disrupted uPA/PAI-1 activity (impairing mNGF generation) and elevated MMP-9 (enhancing its degradation) [29,30]. Other mechanisms likely participate, including potential PFOS-induced upregulation of additional mNGF-metabolizing enzymes. Notably, PFOS has been shown to increase the levels of other MMPs, which mediate the degradation of mNGF [64], so their increase could further promote the observed reduction in mNGF levels. A direct measure of plasminogen levels or MMP-9 activity would allow for a more direct understanding of the alteration in NGF processing. These parameters should be determined in future studies.

THs regulate multiple components of neurotrophic signaling. Studies demonstrate that THs upregulate *NGF* expression in mouse L cells [47] while hypothyroidism increases proNGF and P75^NTR^ and decreases TrkA in developing rat cerebral cortex [20]. The effects of THs extend to other targets: they elevate *PAI-1* expression in T3-L1 adipocytes [48], increase uPA and PAI-1 plasma levels in hyperthyroid patients [49], and specifically raise the PAI-1 plasma levels in subclinical hypothyroidism [50]. THs also upregulate MMP-9 in bone marrow cells [51]. These previous findings collectively support that THs mediated, in part, the regulation of the NGF/TrkA pathway, pointing out that additional mechanisms contribute to these effects. PFOS, known to induce insulin resistance [65], may indirectly affect neurotrophic signaling since insulin regulates *NGF*, *TrkA*, and *P75^NTR^* expression [66]. Insulin resistance also elevates MMP-9 [67] and tPA levels [68]. Furthermore, PFOS increases hepatic HDAC activity in mice [12], and HDAC2 overexpression mimics our observed pattern, increasing *NGF*/proNGF/P75^NTR^ while decreasing mNGF/TrkA [69]. These parallel pathways likely synergize with TH-mediated effects to produce the observed outcomes.

PFOS treatment (1- and 14-day exposures) induces concentration-dependent increases in the content of H_2_O_2_, MDA, and carbonylated proteins, and decreases in NRF2, SOD-1, and HO-1 protein levels (starting at 10 µM or 1 µM, respectively). Co-treatment with NAC completely reversed the effects of PFOS on the studied targets. These results demonstrate that PFOS induces oxidative stress through two main mechanisms: increased production of ROS and impairment of endogenous antioxidant systems. Supporting evidence comes from multiple experimental models. In this sense, single exposure in zebrafish embryos triggered ROS production, lipid peroxidation, and upregulation of the NRF2/HO-1 pathway along with the activation of antioxidant defenses (SOD-1, catalase, glutathione peroxidase) [13]. Similar oxidative effects were observed in rat cerebellar granule cells (3 μM, single exposure) [14] and human SH-SY5Y neuroblastoma cells (50 μM, repeated exposure) [2], where NAC treatment prevented PFOS-induced cytotoxicity. These previous data strongly support our current findings regarding the PFOS oxidative mechanisms and the protective role of NAC. However, we observed opposite effects on the NRF2 pathway, which could be due to exposure duration (initial upregulation as a mechanism of defense and subsequently downregulation due to toxic blockage of the defensive pathway), cell type, or higher sensitivity to TH signaling regulation.

Co-treatment with T3 attenuates but does not completely reverse the PFOS-induced increases in ROS levels, lipid peroxidation, and protein carbonylation as well as the downregulation of the NRF2 antioxidant pathway. These findings demonstrate that T3 protects, in part, from the oxidative stress triggered by PFOS exposure, suggesting, together with our previous studies, that TH signaling disruption contributes to these effects. This aligns with previous studies showing that reduced T3 levels promote oxidative stress by increasing ROS production and suppressing the NRF2 pathway in several brain regions [46,70,71,72]. However, additional mechanisms appear to be involved. In this sense, insulin resistance was described to produce oxidative stress and downregulate the NRF2 pathway [73,74,75,76]. PGE2 has been shown to promote oxidative stress [42,43], and the NGF/TrkA pathway protects against oxidative stress [77]. Thus, PFOS could mediate the oxidative effect observed through PGE2 upregulation and NGF/TrkA pathway disruption, which, in part, is regulated by THs. HDAC2 overexpression was also shown to mediate the production of ROS and weakening of antioxidant defenses [69]. Therefore, these mechanisms may also play a role in mediating these effects.

PFOS treatment (1- and 14-day exposures) induces concentration-dependent increases in COX-2 and PGE2 levels (starting at 10 µM or 1 µM, respectively), with no significant effect on PTGES1 levels. The observed elevation in PGE2 appears to result primarily from increased COX-2 expression, though potential contributions from PTGES1 activity or COX-1 regulation cannot be excluded. These findings align with previous reports showing that repeated PFOS exposure elevates PGE2 in mouse liver [15] and the hepatocarcinoma cell lines [16]. Similarly, acute PFOS treatment increases COX-2 levels in human oral keratinocytes [17], while chronic exposure raises COX-2 in rat jejunal homogenates [18]. Notably, the effects of PFOS on COX-1 and PTGES1 remain unstudied to date.

The PGE2 pathway, NGF signaling, and oxidative stress have been reported to present a bidirectional interaction between them. In this sense, oxidative stress can induce PGE2 production [42], and, in turn, PGE2 can exacerbate oxidative stress [43]. Simultaneously, oxidative stress can alter NGF signaling by affecting TrkA expression and NGF maturation [78], while NGF exerts antioxidant effects through activation of the NRF2/HO-1 pathway [77]. These interrelationships underscore the network nature of neurotoxicity mechanisms, where each component can amplify the others. Future studies should be performed to characterize which of these neurotoxic mechanisms could be the primary cause or if they act in parallel and amplify each other’s response.

Interestingly, co-treatment with T3 attenuates but does not completely reverse PFOS-induced increases in both PGE2 and COX-2, indicating that disruption of thyroid signaling contributes to these effects, though additional mechanisms likely play a role. This is supported by studies showing that THs regulate COX-2 and PGE2 in a tissue-specific manner: hypothyroidism increases COX-2 expression in rat hippocampus [52] while decreasing PGE2 levels in uterine tissue [53]. The opposing effects on PGE2 may reflect tissue-specific responses, species differences, or additional actions of PFOS. Potential mechanisms underlying these effects may involve PFOS-induced overactivation of NMDA receptors [28], which are known to upregulate COX-2 [79] and stimulate PGE2 formation and release [80,81]. Additionally, epigenetic regulation may contribute, as *HDAC2* silencing reduces COX-2 expression [82], and HDAC inhibitors modulate PGE2 levels [83].

Finally, PFOS treatment (1- and 14-day exposures) induces the concentration-dependent cell death of SN56 cells (starting at 10 µM or 1 µM, respectively), which was corroborated by our previous results [28]. PFOS treatment of single *P75^NTR^*-silenced cells or single rNGF, NAC, MF-63, or T3 co-treatment attenuates but does not completely reverse the cell death produced, indicating that all of these mechanisms are involved in the cell death observed. Previous studies showed that NGF/TrkA signaling disruption leads to BFCN loss [84]. ProNGF (increase) or mNGF (decrease) imbalance has been reported to induce cell death [85,86]. P75^NTR^ receptor activation by proNGF triggers BFCN loss [87], but TrkA activation by mNGF is necessary to keep BFCNs alive [86]. The increase in P75^NTR^/TrkA receptors and proNGF/mNGF ratios, as PFOS produced, induces cell death [31,85,86]. Oxidative stress also induces neuronal cell death in BFCNs [46,88]. Therefore, the observed alteration in the P75^NTR^/TrkA ratio is consistent with a shift toward pro-apoptotic signaling, probably together with other pro-apoptotic factors such as oxidative stress, and may contribute to PFOS-induced BFCN death. COX-2 and PGE2 play a well-documented role in neurodegeneration, particularly in BFCN, where its overproduction and activation, respectively, have been linked to neuronal cell death and cognitive impairment [38,39,40,41,89,90]. Additionally, we cannot rule out that the overexpression of COX-2 may lead to an increase in the formation of other prostaglandins such as PGF_2_α, PGD_2_, and TXA_2_, which may also have contributed to neurodegeneration [91], rendering necessary future studies to characterize their relative contribution. THs maintain the BFCN viability [92,93], but their deficiency induces BFCN loss and cognitive decline [92,93,94]. These published findings collectively support our experimental results.

PFOS simultaneous co-treatment with rNGF, NAC, MF-63, and T3 of *P75^NTR^*-silenced cells induced the highest cell death attenuation, but was still incomplete, suggesting that other mechanisms are involved. PFOS single and developmental exposure triggers Aβ and Tau proteins accumulation in SH-SY5Y cells and in adult rat hippocampus [95,96], which were described to produce BFCN loss [46,88]. Aβ activation of P75^NTR^ triggers neuronal apoptosis and contributes to cognitive dysfunction [86,97]. Both insulin resistance and HDAC2 overexpression have also been shown to induce BFCN degeneration [86]. These findings suggest that multiple pathological mechanisms may collectively contribute to the observed neuronal cell death.

PFOS 1- and 14-day treatment induces concentration-dependent effects starting at 10 µM or 1 µM, respectively. The results observed in biological fluids in the human population can reach approximately 0.2 μM, and in the handlers, 10 μM. The results obtained in our study were on a murine model, which is more resistant than a human model, and the concentrations that reach tissue could be higher if we applied the allometric dose conversion model [98]. Therefore, the effects observed for repeated exposures would be within the population’s exposure levels. On the other hand, monitoring levels in biological fluids in the population reflects the remaining amounts of this persistent compound in these individuals. Thus, after a single exposure, the levels reached in tissues will be higher and may exceed 10 μM, as observed in the handlers. According to all of this, we believe that the studied effects are relevant to the human population after single and repeated exposure, since they can be triggered within the range of concentrations that can be reached in the population.

Our data show that PFOS simultaneously induces an imbalance in neurotrophic signaling and severe oxidative stress, both known inducers of apoptosis. While the altered P75^NTR^/TrkA ratio is correlated with cell death, we cannot rule out that oxidative stress acts as a parallel or even synergistic trigger. In fact, the partial protection provided by *P75^NTR^* silencing and by the antioxidant NAC suggests that both mechanisms contribute to the apoptotic outcome. A plausible hypothesis is that PFOS initially activates oxidative stress, which in turn amplifies the neurotrophic imbalance, creating a cycle that culminates in caspase activation. Future experiments with TrkA overexpression during PFOS exposure could isolate the specific contribution of this pathway and determine whether restoring survival signaling is sufficient to block oxidative stress-induced apoptosis. Furthermore, more precise temporal measurements could help establish the sequence of events: whether oxidative stress precedes the neurotrophic receptor alteration or vice versa.

An important consideration in interpreting our data is that apoptosis is an ongoing process during PFOS exposure, leading to substantial protein degradation, while PFOS simultaneously alters the transcription of many genes. This dynamic complicates the distinction between primary, transcription-driven changes in protein levels and secondary changes resulting from caspase-mediated proteolysis. Although our measurements reflect the net protein content (synthesis minus degradation), the relative contributions of transcriptional regulation versus protein turnover cannot be fully disentangled in this experimental design. Future studies are necessary to clarify the precise temporal sequence of molecular events.

Our results indicate that no single treatment (T3, NAC, MF-63, rNGF, or *P75^NTR^* silencing) completely reverses apoptosis, suggesting that neurotoxicity is multifactorial. However, the magnitude of the rescue offers clues to the relative contribution: the antioxidant NAC and the PTGES-1 inhibitor MF-63 produced significantly greater protection than either rNGF administration or *P75^NTR^* silencing alone. This points to oxidative stress and PGE2-mediated inflammation as major drivers of cell death. The even greater protection achieved with upstream T3, and the near-complete protection with the combination of all interventions, supports a model in which disruption of thyroid signaling by PFOS triggers or amplifies multiple cell death pathways that converge on caspase activation.

Our study characterizes the impact of PFOS on discrete neurodegeneration pathways; however, we recognize that the complexity of its interaction with TH pathways, NGF/TrkA/P75^NTR^, and PGE2 signaling, and redox homeostasis suggests the existence of a broader regulatory network. Future research employing systems biology approaches, such as integrating transcriptomic, proteomic, and metabolic profiles with computational network modeling, could identify core nodes and nonlinear relationships that elude reductionist analyses. This integrative framework would allow for a more holistic understanding of PFOS neurotoxicity, revealing potential compensatory mechanisms, cellular resilience thresholds, and pathway synergies, thereby accelerating the identification of multifaceted therapeutic targets.

## 5. Conclusions

In summary, our findings show that PFOS exposure (1- and 14-day treatments) induces BFCN degeneration through multiple interconnected pathways. The observed neurotoxicity is mediated, in part, by PGE2 and NGF/TrkA/P75^NTR^ signaling dysfunction and oxidative stress generation. Notably, our data show that T3 protects, in part, from the effects of PFOS, suggesting that interference in TH signaling contributes to these effects. However, further investigation is required to fully elucidate all molecular mechanisms underlying PFOS neurotoxicity in BFCNs, for in vivo validation of these pathways, and to determine their precise contribution to the cognitive impairments associated with PFOS exposure. These results provide important mechanistic insights into PFOS-induced neurodegeneration and offer potential explanations for the associated learning and memory deficits. From a translational perspective, our work identifies several promising therapeutic targets for protecting against PFOS neurotoxicity, suggesting that combined interventions that simultaneously target multiple pathways offer the greatest therapeutic potential for mitigating PFOS neurotoxicity. However, in vivo preclinical studies are required to validate the efficacy and safety of interventions such as T3, NAC, or COX-2/PGE2 inhibitors in the context of PFOS exposure.

## Data Availability

The original contributions presented in this study are included in the article. Further inquiries can be directed to the corresponding authors.

## Abbreviations

The following abbreviations are used in this manuscript:

Aβ	Amyloid-β
Aβ_1-42_	Amyloid-β Peptide 1-42
ACh	Acetylcholine
AChE	Acetylcholinesterase
AD	Alzheimer’s Disease
ANOVA	Analysis of Variance
BFCNs	Basal Forebrain Cholinergic Neurons
cAMP	Cyclic Adenosine Monophosphate
cDNA	Complementary DNA
COX-1	Cyclooxygenase-1
COX-2	Cyclooxygenase-2
DMEM	Dulbecco’s Modified Eagle’s Medium
DMSO	Dimethyl Sulfoxide
ELISA	Enzyme-Linked Immunosorbent Assay
FBS	Fetal Bovine Serum
HDAC	Histone Deacetylase
HO-1	Heme Oxygenase 1
MDA	Malondialdehyde
MMP9	Matrix Metalloproteinase 9
mNGF	Mature Nerve Growth Factor
MTT	3-(4,5-Dimethylthiazol-2-yl)-2,5-diphenyltetrazolium Bromide
NAC	N-Acetyl Cysteine
NGF	Nerve Growth Factor
NRF2	Nuclear Factor Erythroid 2–Related Factor 2
PAI-1	Plasminogen Activator Inhibitor-1
PBS	Phosphate-Buffered Saline
PFAS	Per- and Polyfluoroalkyl Substances
PFOA	Perfluorooctanoic Acid
PFOS	Perfluorooctane Sulfonic Acid
PPAR-α	Peroxisome Proliferator Activated Receptor A
POPs	Persistent Organic Pollutants
PCBs	Polychlorinated Biphenyls
PGE2	Prostaglandin E2
PTGES1	Prostaglandin E Synthase 1
PGH2	Prostaglandin H2
proNGF	Precursor Nerve Growth Factor
PND	Postnatal Day
ROS	Reactive Oxygen Species
rNGF	Recombinant Nerve Growth Factor
RIPA	Radioimmunoprecipitation Assay Buffer
ruPA	Recombinant Urokinase Plasminogen Activator
SEM	Standard Error of the Mean
siRNA	Small Interfering RNA
SOD-1	Superoxide Dismutase 1
tPA	Tissue Plasminogen Activator
T3	Triiodothyronine
TH	Thyroid Hormone
TIMP-1	Tissue Inhibitor of Metalloproteinases-1
TrkA	Tropomyosin Receptor Kinase A
uPA	Urokinase Plasminogen Activator
